# Validation of a DNA methylation HPV triage classifier in a screening sample

**DOI:** 10.1002/ijc.30008

**Published:** 2016-02-08

**Authors:** Attila T. Lorincz, Adam R. Brentnall, Dorota Scibior‐Bentkowska, Caroline Reuter, Rawinder Banwait, Louise Cadman, Janet Austin, Jack Cuzick, Natasa Vasiljević

**Affiliations:** ^1^ Queen Mary University of London, Centre for Cancer Prevention, Wolfson Institute of Preventive Medicine, Barts and the London School of MedicineCharterhouse SquareLondonEC1M 6BQUnited Kingdom

**Keywords:** HPV, DNA methylation, cervical cancer, triage, biomarkers

## Abstract

High‐risk human papillomavirus (hrHPV) DNA tests have excellent sensitivity for detection of cervical intraepithelial neoplasia 2 or higher (CIN2+). A drawback of hrHPV screening, however, is modest specificity. Therefore, hrHPV‐positive women might need triage to reduce adverse events and costs associated with unnecessary colposcopy. We compared the performance of HPV16/18 genotyping with a predefined DNA methylation triage test (S5) based on target regions of the human gene *EPB41L3*, and viral late gene regions of HPV16, HPV18, HPV31 and HPV33. Assays were run using exfoliated cervical specimens from 710 women attending routine screening, of whom 38 were diagnosed with CIN2+ within a year after triage to colposcopy based on cytology and 341 were hrHPV positive. Sensitivity and specificity of the investigated triage methods were compared by McNemar's test. At the predefined cutoff, S5 showed better sensitivity than HPV16/18 genotyping (74% vs 54%, *P* = 0.04) in identifying CIN2+ in hrHPV‐positive women, and similar specificity (65% vs 71%, *P* = 0.07). When the S5 cutoff was altered to allow equal sensitivity to that of genotyping, a significantly higher specificity of 91% was reached (*P* < 0.0001). Thus, a DNA methylation test for the triage of hrHPV‐positive women on original screening specimens might be a valid approach with better performance than genotyping.

## Abstract 2

**What's new?**

DNA testing for high‐risk human papillomaviruses (hrHPVs) can both detect and predict the development of precancerous cervical lesions. Limitations in specificity, however, necessitate the generation of triage strategies to minimize unneeded colposcopy among hrHPV‐positive women. According to this study, triage may be readily affected using a DNA methylation classifier based on the human gene *EPB41L3* and the late gene regions of HPV16, HPV18, HPV31 and HPV33. The devised classifier outperformed triage by HPV16/18 genotyping in a cohort of hrHPV‐positive patients. The strategy could fill a key role in hrHPV triage in cervical screening programs.

**Abbreviations**
AUC: area under the ROC (receiver operating characteristic) curve
CIN: cervical intraepithelial neoplasia
DNA: deoxyribonucleic acid
hrHPV: high‐risk human papillomavirus
HPV16: human papillomavirus type 16
HPV18: human papillomavirus type 18
HPV31: human papillomavirus type 31
HPV33: human papillomavirus type 33
IQR: interquartile range
P3: predictors 3 study
PCR: polymerase chain reaction
PPV: positive predictive value
qPCR: quantitative polymerase chain reaction
ROC: receiver operating characteristic
S4: DNA methylation classifier score 4
S5: DNA methylation classifier score 5

Human papilloma virus (HPV) infection is very common worldwide; however, most episodes are transient and persistence beyond 2 years with high‐risk (hr) types occurs in <10% of women.^1^ Persistent hrHPV infection drives development of high‐grade cervical intraepithelial neoplasia (CIN2 or CIN3) which may, if left untreated, progress to invasive cancer. Evidence that hrHPV testing is more sensitive than cytology^2^, ^3^, ^4^, ^5^ has driven implementation of the American Society for Colposcopy and Cervical Pathology recommendation^6^ to use reflex hrHPV testing as a triage to colposcopy in women who present with abnormal cytology in high‐income regions. Recent evidence also suggests that a primary hrHPV screening test could provide better protection against cancer risk than cytology,^7^ because it identifies almost all prevalent CIN2+ as well as those at risk of CIN2+.^8^, ^9^ However, an important drawback of HPV screening is its modest specificity and positive predictive value (PPV), creating a need for triage to minimize unneeded referrals to colposcopy. Previous proposals for the triage of hrHPV‐positive women include Papanicolaou cytology, genotyping for HPV16 and HPV18, and immunostaining for p16, with or without ki‐67. However, these methods have important limitations, including a relatively low sensitivity, low PPV, and subjectivity.^10^

Measuring DNA methylation at specific CpG sites in HPV or human genes has shown promise for the accurate detection of CIN2+.^11^, ^12^, ^13^, ^14^, ^15^, ^16^ Moreover, cervical cancers nearly always show high levels of gene methylation.^17^, ^18^ It is the late HPV capsid genes (L1 and L2) that exhibit greatest difference in methylation between women diagnosed with CIN2+ and those with normal or a mild lesion and the increase in methylation is in direct relation to increasing lesion severity.^12^, ^13^, ^14^ The levels of methylation also increase over time in women with persistent HPV16 infection regardless of prevalent CIN.^19^, ^20^ Among a plethora of suggested human biomarker genes, methylation of the promoter or introns of *CADM1, MAL, EPB41L3, TERT, PAX1, SOX1* and *LMX1* have shown promise for clinical utility.^21^, ^22^, ^23^ Methylation of human genes also increase with length of HPV persistence, and elevated methylation may be detected up to 7 years before discovery of a cancer.^13^ Therefore, accurate measurement of DNA methylation may be useful for triage in HPV‐based screening programs, by helping to identify women who would develop cervical cancer if untreated.

We have developed a triage classifier called S5 based on DNA methylation of the late regions of HPV16, HPV18, HPV31 and HPV33 combined with the promoter region of a human gene *EPB41L3*.^24^ The main objective of this study was to assess the use of S5 as a triage test to identify CIN2+ in hrHPV‐positive women from a London screening cohort,^25^ and to compare it with HPV16/18 genotyping. The secondary aim was to compare the performance of S5 with an earlier risk score (S4) that does not use HPV33 methylation.^24^, ^26^

## Material and Methods

### Patients

**Figure 1:**
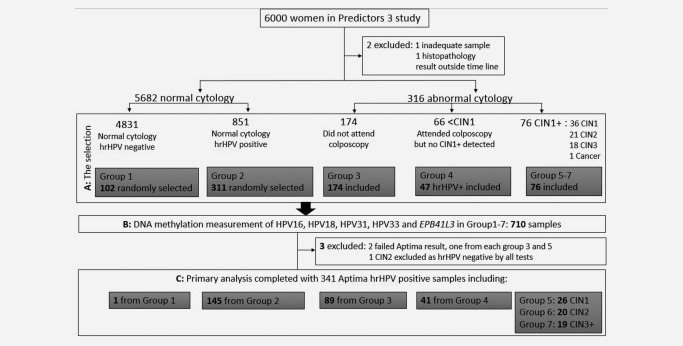
Flowchart describing the selection and analysis of the 7 groups from the P3 screening population. Abnormal cytology result encompasses borderline or worse. (A) For selection of hrHPV‐positive samples, the combined results of the BD and Abbott tests were considered and a sample was positive if either test identified it as such. In summary, 413 women with normal cytology (Group 1–2) were randomly selected while we included all women with abnormal cytology (Group 3–7) except for 19 hrHPV‐negative women with <CIN1 confirmed on colposcopy (group 4). (B) DNA methylation was measured in all 710 selected samples regardless of HPV genotype results. (C) 367 Aptima HPV‐test‐negative samples were excluded from the primary analysis and remaining samples in each group are indicated.

This study was conducted following REMARK guidelines for assessing biomarker test performance.^27^ Residual material from liquid‐based cytology PreservCyt was obtained from 6000 women attending for routine screening in London UK (Fig. 1). Full details of the Predictors 3 (P3) study, which investigated the performance of several different HPV nucleic acid tests, have been reported.^25^ The main clinical endpoint was histology result within 12 months of the abnormal smear. CIN status was based on local histopathology, taking the highest grade of abnormality seen in the biopsy or treatment specimen.

We selected 710/6000 (12%) women from P3 by sampling groups based on hrHPV positivity, cytology results and CIN status (Fig. 1). For the selection, hrHPV positivity was defined by combination of an Abbott RealTime High Risk HPV assay (Abbott Molecular GmbH & Co. KG, Wiesbaden, Germany) and a BD HPV test (Becton Dickinson Diagnostics, Sparks, Maryland, USA), where the hrHPV positives were defined to be positive by either of these tests. The BD and Abbott test provided HPV16/18 genotyping individually, while HPV31 genotyping and a pooled result for HPV33 (along with types 56, 58 and 66) were only available from the BD test. The genotyping information was used for quality control of the methylation assay. For the primary analysis, Aptima (Hologic Inc, San Diego, CA, USA) result was used to determine hrHPV status, which we further describe in the statistical methods.

The P3 study was approved by the Imperial NHS Trust Tissue Management Committee and the Multicentre Research Ethics Committee for Wales. Individual consent was not required as the study was noninvasive and used screening residual samples which would otherwise be discarded. The identities of the women were fully anonymized and identifiable to the research team only by subject number. Cytology and histopathology data were linked to the HPV result by the center and then all data were anonymized before release to the research team.

### The methylation assays

DNA was extracted from aliquots of the liquid‐based cytology samples with the QIAamp DNA Mini Kit (Qiagen Inc, Hilden, Germany). Two hundred and fifty nanograms of DNA was used in the bisulfite conversion reactions, where unmethylated cytosines were converted to uracil with the EZ DNA methylation kit (Zymo research, Irvine, USA). Converted DNA from an equivalent of 1600 cells per sample were amplified by methylation‐independent PCR primers and the amplicons were tested in triplicate by pyrosequencing for DNA methylation of *EPB41L3* and the late (L1 and L2) regions of HPV16, HPV18 and HPV31 and HPV33, as detailed previously.^14^, ^15^ The laboratory was blinded to cytology, histology and HPV test results; therefore, each methylation assay encompassed in the S5 classifier was run on all selected specimens. Percentage methylation was taken as the mean from the triplicate results.

### Statistical methods

The primary clinical end point was CIN2+, and the main aim was to validate the performance of the S5 classifier in comparison with HPV16/18 genotyping in hrHPV‐positive women. HPV positivity in the statistical analysis was determined by Aptima HPV test because it previously showed the highest sensitivity and specificity.^25^ Therefore, this was the most rigorous comparison possible and meant that any apparent improvements produced by measurement of methylation were unlikely to be confounded by the level of accuracy of the HPV test. S5 was compared to the genotyping data obtained from Abbott test as this information was not supplied by the Aptima test.

S5 was defined as S5 = 30.9(*EPB41L3)* + 13.7(HPV16_L1_)+ 4.3(HPV16_L2_) + 8.4(HPV18_L2_) + 22.4(HPV31_L1_) + 20.3 (HPV33_L2_) with individual CpG sites described previously.^24^, ^26^ Sensitivity and specificity at a predefined cutpoint S5 = 0.8, which attained >90% sensitivity in the previous study, was used for the main comparison.^24^ We also compared the difference in specificity at a cutpoint, where the sensitivity was equal to the HPV16/18 genotyping.

Secondary analysis considered an earlier risk of CIN2+ score, S4, that did not include HPV33 methylation: S4 = 38.8(*EPB41L3)* + 17.2(HPV16_L1_) + 5.4(HPV16_L2_) + 28.1(HPV31_L1_) + 10.5(HPV18_L2_) with a triage cutpoint S4 = 0.5.^26^

Wilson confidence intervals were used for the primary outcomes of sensitivity, specificity and PPV at cut points; McNemar's test with continuity correction was used for differences in sensitivity and specificity.^28^ The performance of continuous risk scores was measured by area under the curve (AUC) with a Wilcoxon test and DeLong confidence intervals.^29^ A likelihood‐ratio test was used for the differences between continuous risk scores. All *P*‐values were two sided. Analyses were undertaken using the software GNU R 2.15.1.^30^

## Results

### S4 and S5 methylation classifier in the P3 sample cohort

We successfully measured *EPB41L2* methylation in 707/710 of the selected P3 samples. The HPV methylation assay amplified and detected 99 samples as positive for HPV16, 36 for HPV18, 55 for HPV31 and 43 for HPV33. These HPV methylation‐positive samples were in >89% agreement with BD and Abbott genotyping data (Supporting Information, Table 1).

**Figure 2:**
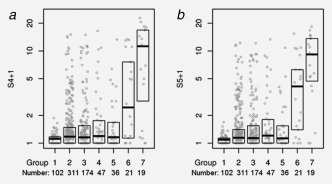
Distribution of (a) S4 and (b) S5 by population group that was sampled. The median and interquartile range are depicted by boxes and the individual scores by grey circles. Groups 1–7 correspond to the groups described in Figure 1, where Group 5 through 7 represent 36 CIN1, 21 CIN2 and 19 CIN3+, respectively.

The S4 and S5 value was calculated for each sample, by inserting the methylation values into our predefined classifier score equations. The distribution of the scores within the 7 groups sampled is shown in Figure 2. A Cuzick test for trend confirmed significantly increasing methylation with group number for S4 $\chi \frac{2}{1}=$ 38.5 (*P* < 0.0001) and a significantly larger trend for S5 $\chi \frac{2}{1}=$ 55.9 (*P*_diff_ < 0.0001). There was one cancer in the study which was included in both the CIN2+ and CIN3+ analyses. This sample was HPV16 positive with high methylation levels in the viral genes (top 2%) as well as *EPB41L3* (top 1%).

### Methylation versus genotyping

Out of 710 samples, 341 were positive for hrHPV by the Aptima test including 146 women with normal cytology (Group 1–2), 89 women with abnormal cytology who did not attend colposcopy (Group 3), 41 women with abnormal cytology and <CIN1 on colposcopy (Group 4) and 65 women CIN1+ (Fig. 1). One CIN2 in the study was omitted from the primary analysis because it was hrHPV negative by Aptima and all other HPV DNA tests including methylation tests. The women were aged between 20 and 64 years and the mean age difference was only 0.06 years between the hrHPV‐positive <CIN2 and CIN2+ (*P* = 0.26).

To assess which method would be more effective to triage women to colposcopy following a hrHPV‐positive test result, Abbott HPV16/18 genotyping data was compared to the S5 classifier at a predefined cutpoint (Table 1). The S5 classifier showed significantly higher sensitivity (McNemar *χ* 2 = 4.08, *P* = 0.043) and similar specificity (*χ* 2 = 3.21, *P* = 0.07) to HPV16/18 genotyping (Table 1). A cross‐tabulation of the classifiers by CIN2+ status is presented in Supporting Information, Table 2. In addition, the same comparison was performed using either the Abbott or BD test to define the hrHPV positivity; this further confirmed that S5 methylation performed with significantly higher sensitivity and no change in specificity irrespective of the HPV test (Supporting Information, Table 3). The two triage methods were also compared by adjusting the cutpoint for S5 to obtain the same sensitivity as genotyping. This revealed significantly better specificity of S5 at 91% (95 CI 87–94) (McNemar *χ* 2 = 52.17, *P* > 0.0001).

**Table 1:** Comparison of triage rules in 341 Aptima hrHPV‐positive women using either HPV16/18 genotyping or DNA methylation measurement according to classifiers S4 or S5

		HPV16/18	S5	S4
Sensitivity (95% CI)	CIN3+^a^	0.58 (0.36–0.77)	0.84 (0.62–0.94)	0.74 (0.51–0.88)
Specificity (95% CI)		0.69 (0.64–0.74)	0.63 (0.58–0.68)	0.59 (0.53–0.64)
Sensitivity (95% CI)	CIN2+	0.54 (0.39–0.68)	0.74 (0.59–0.85)	0.69 (0.54–0.81)
Specificity (95% CI)		0.71 (0.65–0.75)	0.65 (0.60–0.70)	0.59 (0.53–0.64)

a In the analysis with the CIN3+ endpoint, the CIN2 were excluded as we did not wish to include these lesions with <CIN2.

Predefined cut points were applied to S5 (0.8) and S4 (0.5). Number of patients with positive and negative test results for each test is reported in Supporting Information, Table 4.

Investigating the reason behind the superior performance of S5, a univariate analysis of each component showed that *EPB41L3* and HPV16 and HPV33 methylation in women who tested positive for these types gave substantial additional information (Table 2). Although HPV18 and HPV31 were not individually significant, this was probably due to lack of power.

**Table 2:** Summary statistics for the individual CpG sites and the components of S5

	N^a^	CIN2+	CIN3+	AUC^b^ (CIN2+)	*P* (CIN2+)	AUC^b^ (CIN3+)	*P* (CIN3+)
16:6367	87	21	6	0.69	8.0e‐03	0.78	3.2e‐03
16:6389	87	21	6	0.73	1.3e‐03	0.85	2.3e‐04
16L2:4275	87	21	6	0.72	6.0e‐04	0.75	2.6e‐03
16L2:4268	87	21	6	0.69	8.1e‐03	0.74	8.2e‐03
16L2:4259	87	21	6	0.69	4.9e‐03	0.75	3.9e‐03
16L2:4247	87	21	6	0.62	7.7e‐02	0.66	6.9e‐02
16L2:4238	87	21	6	0.74	9.1e‐04	0.86	9.6e‐05
31:6352	44	3	0	0.67	3.5e‐01	0.68	5.6e‐01
31:6364	44	3	0	0.73	1.9e‐01	0.80	3.2e‐01
18:4256	24	1	1	0.09	1.9e‐01	0.09	1.9e‐01
18:4261	24	1	1	0.96	1.5e‐01	0.96	1.5e‐01
18:4265	24	1	1	0.83	3.1e‐01	0.83	3.1e‐01
18:4269	24	1	1	0.70	5.6e‐01	0.70	5.6e‐01
18:4275	24	1	1	0.78	3.8e‐01	0.78	3.8e‐01
18:4282	24	1	1	0.07	1.7e‐01	0.07	1.7e‐01
33:5557	34	9	3	0.64	2.0e‐01	0.88	6.5e‐02
33:5560	34	9	3	0.71	6.4e‐02	1.00	2.2e‐02
33:5566	34	9	3	0.76	2.4e‐02	0.98	2.8e‐02
33:5572	34	9	3	0.62	3.0e‐01	0.98	2.6e‐02
EPBL143 (x_1_ c)	341	39	19	0.73	8.1e‐07	0.80	3.6e‐06
HPV16‐L1 (x_2_)	341	39	19	0.69	3.5e‐07	0.72	8.2e‐06
HPV16‐L2 (x_3_)	341	39	19	0.67	1.5e‐07	0.73	1.7e‐07
HPV31 (x_4_)	341	39	19	0.47	3.7e‐01	0.46	3.3e‐01
HPV18 (x_5_)	341	39	19	0.48	2.8e‐01	0.49	7.6e‐01
HPV33 (x_6_)	341	39	19	0.59	2.5e‐04	0.52	4.1e‐01

*P* values were calculated from a Wilcoxon test.

a *N* shows the total number of samples in each analysis of histopathological endpoints of interest.

b AUC = area under the curve.

c x_1_ to x_6_ indicate the combined component variables (expressed as mean methylation) in the classifiers;.

### S4 versus S5 methylation classifier

**Figure 3:**
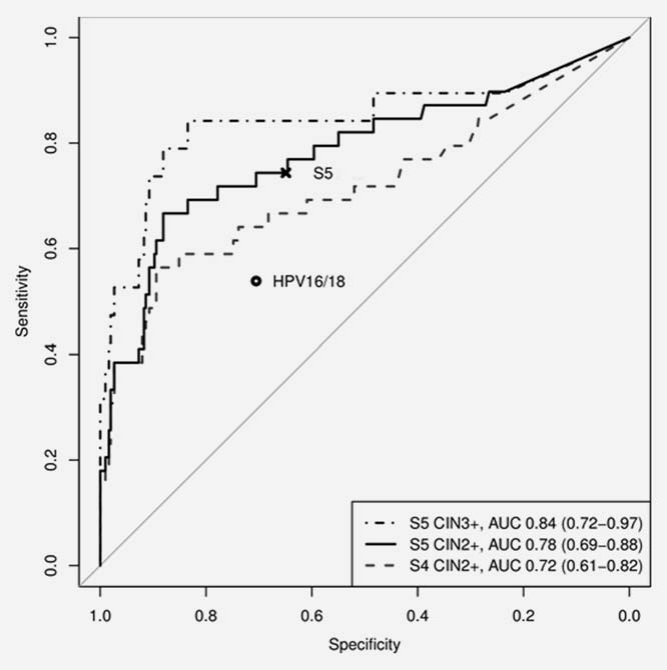
Receiver operator characteristic plots for S4 and S5. The (x) denotes the sensitivity and specificity at the S5 cut‐point 0.8 and as a comparison, the HPV16/18 genotyping point result is pictured by (o).

We compared the performance of the S4 to the S5 classifier. Although S4 had comparable sensitivity 69% (Table 1) (*χ* ^2^ = 0.17, *P* = 0.68) to S5, poorer specificity 59% was observed (*χ* 2 = 7.90, *P* = 0.0049). A comparison of the receiver operator characteristic (ROC) curves showed that S5 had an AUC of 0.78 (95% CI 0.69–0.88) versus 0.72 (95% CI 0.61–0.82) for S4 (Δ*χ* 2 = 17.5, *P* < 0.0001) (Fig. 3). There was an increasing trend of methylation and scores from CIN2 to CIN3 (Table 1 and Fig. 2), so most of the measures were improved for CIN3+.

### hrHPV positive versus hrHPV negative

**Figure 4:**
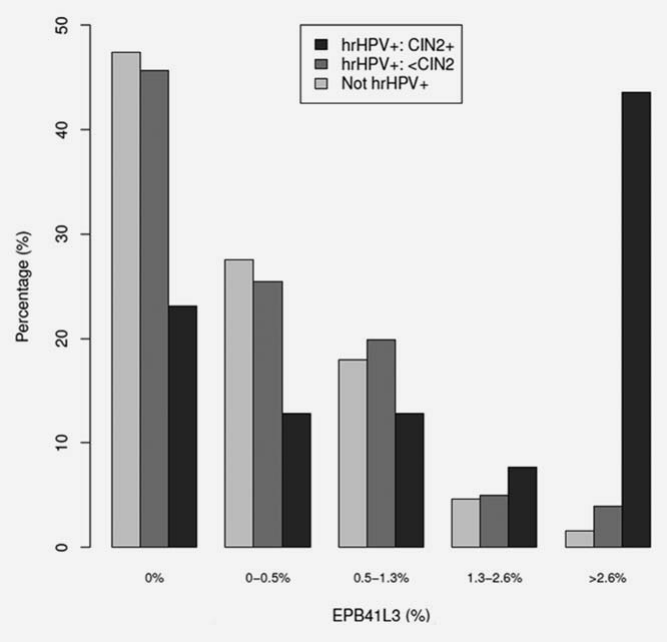
Percentage distribution of *EPB41L3* methylation by hrHPV positivity and CIN status in 710 P3 patients. Of particular interest was a methylation cut‐point for *EPB41L3* when a sample was not positive for HPV16, 18, 31 or 33; or unmethylated if positive, which was simply calculated at the predefined cutpoints for S5 and S4 classifiers as S5 = 0.8/30.9 ≈ 2.6% and S4 = 0.5/38.8 ≈ 1.3%, respectively.

Finally, to assess if there was a significant difference between hrHPV‐negative and hrHPV‐positive women stratified by CIN status, we considered the methylation of human gene *EPB41L3* in all samples. Figure 4 shows that there was very little difference between the <CIN2 hrHPV‐positive and the hrHPV‐negative samples (*P* = 0.24).

## Discussion

We validated a DNA methylation classifier of CIN2+ histology, using hrHPV‐positive women from a UK screening group. The AUC obtained in this study was 0.78 (95% CI 0.69–0.88) compared with 0.82 (95% CI 0.80–0.84) in the colposcopy referral sample originally used to develop the classifier.^24^ The S5 classifier is a multibiomarker panel composed of a human gene *EPB41L3* and the late regions of the four clinically most important HPV types: HPV16, HPV18, HPV31 and HPV33.^31^, ^32^ S5 performed significantly better than an alternative methylation classifier (S4) that lacked measurement of HPV33.^24^, ^26^ We further observed that the HPV33 component was more important than either HPV18 or HPV31 (Table 2). The four main randomized controlled trials investigating the efficacy of hrHPV testing as a primary screen compared cytology with cytology combined with hrHPV testing.^8^ Although cytology is the most likely test to be used secondary to an hrHPV test, due to the design of our study, we were unable to compare the methylation classifiers to cytology. Furthermore, we were interested in evaluating a fully molecular test, avoiding the complications with specimen requirements and processing associated with the use of cytology. Therefore, we compared the S5 classifier to the most common molecular triage approach, which is already available as a reflex test from several manufacturers, namely, genotyping for HPV16 and HPV18. Here, we showed that two methylation classifiers outperformed genotyping for HPV16/18. It is possible that in future, expanded genotyping for all 14 individual types may be shown to have additional clinical value; however, we were not able to compare our methylation classifiers to expanded genotyping because of lack of availability of the data and because there were too few CIN2+ to allow a meaningful comparison for the less prevalent hrHPV types. At the predefined cutoff, S5 had a better sensitivity than triage using HPV16/18 genotyping, and shows promise as a triage test for hrHPV‐positive women. It is likely that an adjustment of the cutoff may be needed to accommodate the difference between screening and colposcopy referral populations. If we allowed that adjustment and compared the two methods by equalizing the sensitivity to that of HPV16/18 genotyping (54%), the specificity of S5 reached 91% and was significantly higher than that of genotyping (*P* < 0.0001) further confirming the advantage of methylation testing compared to genotyping.

Earlier studies have shown that cervical cancers have higher levels of methylation than CIN3, suggesting the possibility that methylation may be used to indicate the CIN2/3 destined to progress from those that will regress or remain as indolent CIN2/3 lesions.^17^, ^18^, ^21^ Concurrently, there was one cervical cancer in our study, which was positive for HPV16, and it had very high methylation for both HPV16 L1 and for *EPB41L3*.

In addition, we compared if methylation of *EPB41L3* was different in hrHPV‐positive and ‐negative women, but observed very little difference (Fig. 4). In light of these results, it is possible to envisage a screening test that simultaneously genotypes and measures methylation levels of HPVs and *EPB41L3*. Such fully integrated molecular screening‐triage tests would provide the benefit of immediate and more accurate results that separate women into three management groups: (i) negative for all biomarkers, who would go back to routine screening; (ii) hrHPV‐positive and methylation‐negative, who would have repeat testing and (iii) methylation positive regardless of hrHPV status, who would be referred to colposcopy. Other uses of DNA methylation testing may be a triage to clinical attention for women who choose to provide vaginal self‐samples instead of attending cervical screening programs. In a recent report, triage by DNA‐methylation test was shown noninferior to cytology for detection of CIN2+.^16^

The strength of this study is the validation of the S5 classifier in a routine screening study in the UK with blinding of all results to the lab technicians, and the use of prespecified cutoffs for the methylation classifiers which minimized the risks for bias and overfitting. In practice, hrHPV‐positive women could have the methylation tests performed on the original samples in a reflex manner, triaging women at risk to colposcopy and thereby reducing anxiety and overtreatment in the low‐risk women. Possible concerns over missing some of the CIN2 and CIN3 might be addressed by referring women negative or low risk by the DNA methylation classifier to repeat HPV testing in 1 year. It is plausible that prospective studies will show low or negative methylation test results to indicate certain CIN2/3 that are unlikely to progress.^13^ Indeed, most CIN2 have been shown to regress and while only a minority of CIN3 regress, most persist indolently with only a small fraction progressing to cancer in any given year.^33^ Women with long‐term persisting low‐risk CIN3 can be detected in later rounds of screening and treated based on clinical judgement. Large long‐term prospective studies are needed to clarify these issues of CIN2/3 progression and regression.

A limitation of our analysis is that women with normal cytology who may have had occult CIN2+ were classified as <CIN1 in our cohort because referral to colposcopy did not consider the HPV DNA results. To address this, we restricted an analysis to include only 41 hrHPV‐positive women who were confirmed <CIN1 by colposcopy as controls, but this made no difference and only confirmed the finding of our primary analysis (Supporting Information, Fig. 1 and Table 4). To further address this issue, future validation work is planned in studies, where all hrHPV‐positive women are referred to colposcopy. Another possible group with occult CIN2+, which was included here, was Group 3—the 89 Aptima hrHPV‐positive women, who had borderline, mild and moderate dyskaryosis cytology result but who did not attend colposcopy (Fig. 1). A subgroup analysis excluding Group 3 showed only minor difference in sensitivity and specificity (Supporting Information, Table 2).

All hrHPV‐positive women in P3 were not included in this study, which can be also viewed as a limitation. The fact that hrHPV‐positive women who had normal or occasional borderline cytology were not followed up is a drawback but this works against the methylation classifier because fewer CIN2+ are predicted to be discovered with inadequate follow‐up and this has the effect of making the specificity and PPV of the methylation test lower than it would be in the absence of verification bias. More work is needed to help address the issues that these questions raise for triage and screening.

We conclude that DNA methylation triage of hrHPV‐positive women on original screening specimens may be regarded as validated and may offer improved workflows compared to cytology and better performance than HPV16/18 genotyping. It is therefore important to further test our triage S5 model in large prospective studies.

## Supporting information

Supporting Information
