# Supplementary material for: Validation of a DNA methylation HPV triage classifier in a screening sample
Source: Int J Cancer. 2016 Feb 8;138(11):2745–51. doi: 10.1002/ijc.30008 (PMC4832297; doi:10.1002/ijc.30008)
Supplement: Supplementary file 1 — Supporting Information [file IJC-138-2745-s001.doc]

Supplemental Table 1. The number of samples in which methylation was successfully measured and the percentage in agreement with the BD or Abbott genotyping test.

|  | BD genotype positive | Abbott genotype positive |
| --- | --- | --- |
| HPV16 methylation measured (n=99) | 96% (n=95) | 95% (n=94) |
| HPV18 methylation measured (n=36) | 89% (n=32) | 92% (n=33) |
| HPV31 methylation measured (n=55) | 95% (n=52) | N/A |
| HPV33 methylation measured (n=43) | N/A | N/A |

Supplemental Table 2. Cross-tabulation of classifiers.

| Comparison |  | Test 1 / Test 2 result | | | |
| --- | --- | --- | --- | --- | --- |
| Test 1 | Test 2 | - / - | -/+ | +/- | +/+ |
| *CIN2*+ |  |  |  |  |  |
| HPV16/18 | S4 | 11 (28%) | 7 (18%) | 1 (3%) | 20 (51%) |
| HPV16/18 | S5 | 8 (21%) | 10 (26%) | 2 (5%) | 19 (49%) |
| S4 | S5 | 8 (21%) | 4 (10%) | 2 (5%) | 25 (64%) |
| *< CIN2* |  |  |  |  |  |
| HPV16/18 | S4 | 169 (56%) | 44 (15%) | 9 (3%) | 80 (26%) |
| HPV16/18 | S5 | 170 (56%) | 43 (14%) | 27 (9%) | 62 (21%) |
| S4 | S5 | 167 (55%) | 11 (4%) | 30 (10%) | 94 (31%) |

Supplemental Table 3. Comparison of triage rules in 437 Abbott or 515 BD hrHPV positive women using either HPV16/18 genotyping or DNA methylation measurement according to classifiers S4 or S5. Predefined cut points were applied to S5 (0.8) and S4 (0.5).

|  |  |  | HPV16/18 | S5 | S4 |
| --- | --- | --- | --- | --- | --- |
|  | Sensitivity (95% CI) | CIN3+^a^ | 0.61 (0.39-0.80) | 0.89 (0.67-0.97) | 0.78 (0.55-0.91) |
| Abbott | Specificity (95% CI) |  | 0.69 (0.64-0.73) | 0.70 (0.66-0.74) | 0.64 (0.59-0.68) |
|  | Sensitivity (95% CI) | CIN2+ | 0.55 (0.40-0.70) | 0.76 (0.61-0.87) | 0.71 (0.55-0.83) |
|  | Specificity (95% CI) |  | 0.70 (0.65-0.74) | 0.70 (0.66-0.74) | 0.64 (0.59-0.68) |
|  | Sensitivity (95% CI) | CIN3+^a^ | 0.58 (0.36-0.77) | 0.84 (0.62-0.94) | 0.74 (0.51-0.88) |
|  | Specificity (95% CI) |  | 0.75 (0.71-0.78) | 0.74 (0.70-0.78) | 0.68 (0.63-0.72) |
| BD | Sensitivity (95% CI) | CIN2+ | 0.54 (0.39-0.68) | 0.74 (0.59-0.85) | 0.69 (0.54-0.81) |
|  | Specificity (95% CI) |  | 0.76 (0.72-0.79) | 0.74 (0.70-0.78) | 0.68 (0.63-0.72) |

1. In the analysis with the CIN3+ endpoint the CIN2 were excluded as we did not wish to include these lesions with <CIN2.

Supplemental table 4. Comparison of triage rules at predefined cut points, S5 (0.8) and S4 (0.5) with the Abbott HPV16/18 genotyping in 206 Aptima hrHPV+ women. The <CIN1 control group comprises only the 41 women who attended colposcopy.

|  | Endpoint | S5 (cut 0.8) | HPV16/18 | S4 (cut 0.5) |
| --- | --- | --- | --- | --- |
| Sensitivity (95% CI) | CIN2+ | 0.74 (0.59-0.85) | 0.54 (0.39-0.68) | 0.69 (0.54-0.81) |
| Specificity (95% CI) |  | 0.70 (0.58-0.80) | 0.79 (0.68-0.87) | 0.61 (0.49-0.72) |
| Sensitivity (95% CI) | CIN3+ | 0.84 (0.62-0.94) | 0.58 (0.36-0.77) | 0.74 (0.51-0.88) |
| Specificity (95% CI) |  | 0.62 (0.52-0.72) | 0.72 (0.62-0.81) | 0.61 (0.49-0.72) |

Supplemental table 5. The number of test positive in each diagnostic group at predefined cut points, S5 (0.8) and S4 (0.5) with the Abbott HPV16/18 genotyping. The numbers are compared in two different parameter settings: 1) when all <CIN were included and 2) when <CIN1 control group comprised only the 41 women who attended colposcopy.

| Diagnosis | Test result | S5 (cut 0.8) | | HPV16/18 | | S4 (cut 0.5) | |
| --- | --- | --- | --- | --- | --- | --- | --- |
|  |  | 1) | 2) | 1) | 2) | 1) | 2) |
| <CIN1 | - | 29 | 179 | 34 | 194 | 27 | 164 |
|  | + | 12 | 97 | 7 | 82 | 14 | 112 |
| CIN1 | - | 18 | 18 | 19 | 19 | 14 | 14 |
|  | + | 8 | 8 | 7 | 7 | 12 | 12 |
| CIN2 | - | 7 | 7 | 10 | 10 | 7 | 7 |
|  | + | 13 | 13 | 10 | 10 | 13 | 13 |
| CIN3 | - | 3 | 3 | 8 | 8 | 5 | 5 |
|  | + | 15 | 15 | 10 | 10 | 13 | 13 |
| Cancer | - | 0 | 0 | 0 | 0 | 0 | 0 |
|  | + | 1 | 1 | 1 | 1 | 1 | 1 |


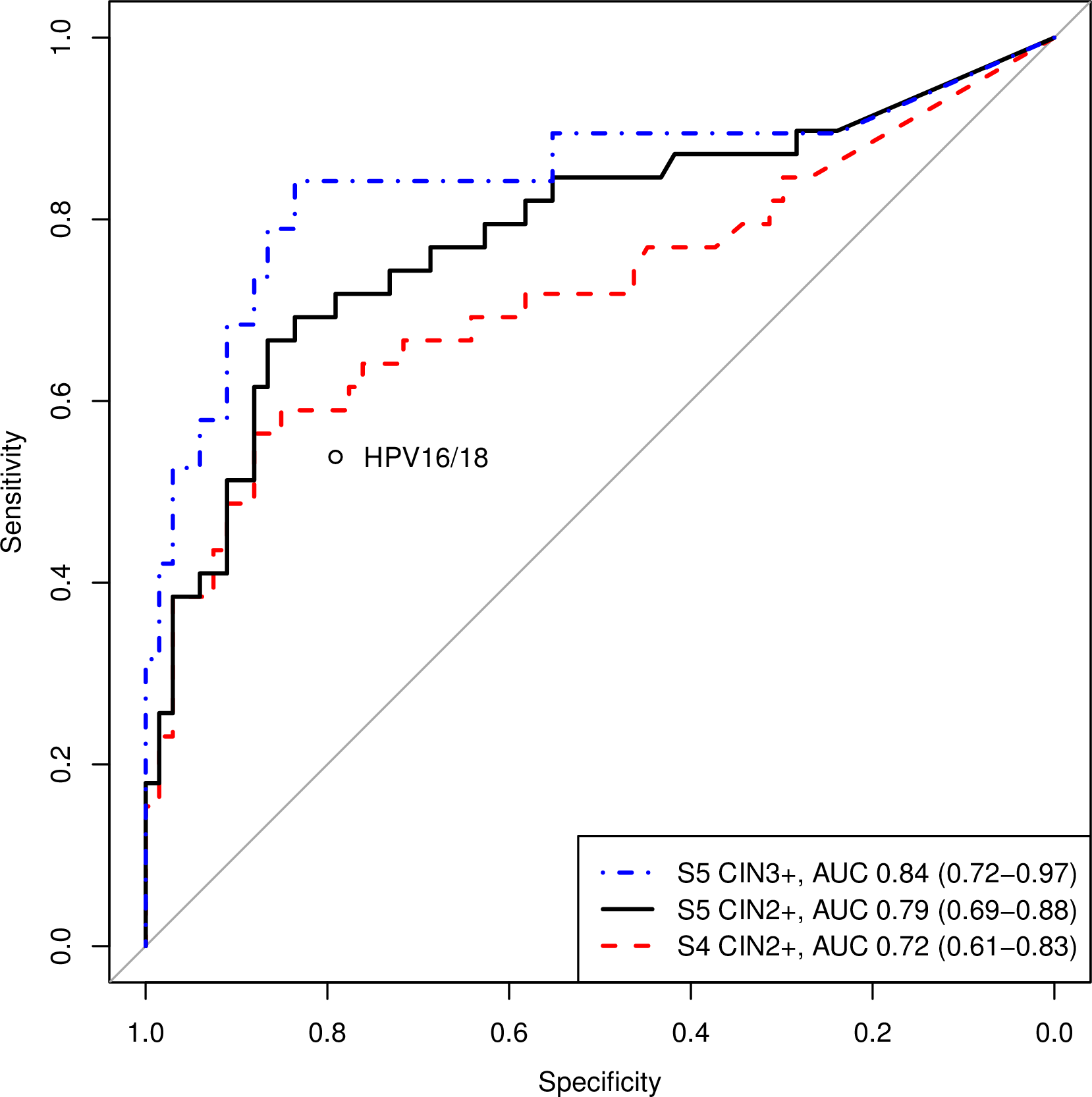


Supplemental figure 1. Receiver operator characteristic plots for S4 and S5 where 41 Aptima hrHPV+ women who attended colposcopy but were found <CIN1 comprise the control group. The HPV16/18 genotyping point result (o) is shown for comparison.
